# Redetermination and absolute configuration of atalaphylline

**DOI:** 10.1107/S160053680905449X

**Published:** 2010-01-09

**Authors:** Hoong-Kun Fun, Chin Sing Yeap, Suchada Chantrapromma

**Affiliations:** a X-ray Crystallography Unit, School of Physics, Universiti Sains Malaysia, 11800 USM, Penang, Malaysia; b Crystal Materials Research Unit, Department of Chemistry, Faculty of Science, Prince of Songkla University, Hat-Yai, Songkhla 90112, Thailand

## Abstract

The title acridone alkaloid [systematic name: 1,3,5-trihydr­oxy-2,4-bis­(3-methyl­but-2-en­yl)acridin-9(10*H*)-one], C_23_H_25_NO_4_, has previously been reported as crystallizing in the chiral ortho­rhom­bic space group *P*2_1_2_1_2_1_ [Chantrapromma *et al.* (2010 ▶). *Acta Cryst.* E**66**, o81–o82] but the absolute configuration could not be determined from data collected with Mo radiation. The absolute configuration has now been determined by refinement of the Flack parameter with data collected using Cu radiation. All features of the mol­ecule and its crystal packing are similar to those previously described.

## Related literature

For details of acridone alkaloids see: Basu & Basa (1972 ▶). For the previous structure determination, see: Chantrapromma *et al.* (2010 ▶). For hydrogen-bond motifs, see Bernstein *et al.* (1995 ▶). For bond-length data, see: Allen *et al.* (1987 ▶). For the stability of the temperature controller used in the data collection, see Cosier & Glazer, (1986 ▶).
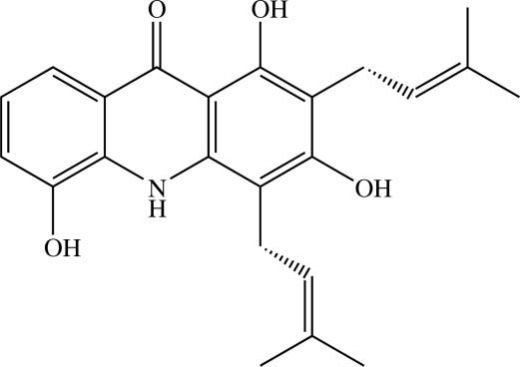


## Experimental

### 

#### Crystal data

C_23_H_25_NO_4_
*M* *_r_* = 379.44
Orthorhombic, 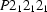

*a* = 5.0838 (1) Å
*b* = 15.0262 (3) Å
*c* = 24.6412 (4) Å
*V* = 1882.35 (6) Å^3^
*Z* = 4
Cu *K*α radiation
μ = 0.74 mm^−1^
*T* = 150 K
0.40 × 0.21 × 0.04 mm

#### Data collection

Bruker APEX Duo CCD area-detector diffractometer
Absorption correction: multi-scan (*SADABS*; Bruker, 2009 ▶) *T*_min_ = 0.755, *T*_max_ = 0.970
11768 measured reflections
3145 independent reflections
3099 reflections with *I* > 2σ(*I*)
*R*_int_ = 0.017

#### Refinement

*R*[*F* ^2^ > 2σ(*F* ^2^)] = 0.025
*wR*(*F* ^2^) = 0.068
*S* = 1.06
3145 reflections
354 parameters
All H-atom parameters refined
Δρ_max_ = 0.12 e Å^−3^
Δρ_min_ = −0.10 e Å^−3^
Absolute structure: Flack (1983 ▶), 1280 Friedel pairs
Flack parameter: 0.05 (13)

### 

Data collection: *APEX2* (Bruker, 2009 ▶); cell refinement: *SAINT* (Bruker, 2009 ▶); data reduction: *SAINT*; program(s) used to solve structure: *SHELXTL* (Sheldrick, 2008 ▶); program(s) used to refine structure: *SHELXTL*; molecular graphics: *SHELXTL*; software used to prepare material for publication: *SHELXTL* and *PLATON* (Spek, 2009 ▶).

## Supplementary Material

Crystal structure: contains datablocks global, I. DOI: 10.1107/S160053680905449X/sj2714sup1.cif

Structure factors: contains datablocks I. DOI: 10.1107/S160053680905449X/sj2714Isup2.hkl

Additional supplementary materials:  crystallographic information; 3D view; checkCIF report

## Figures and Tables

**Table 1:** Hydrogen-bond geometry (Å, °)

*D*—H⋯*A*	*D*—H	H⋯*A*	*D*⋯*A*	*D*—H⋯*A*
O1—H1*O*1⋯O2	0.915 (19)	1.699 (19)	2.5528 (13)	154.1 (17)
O3—H1*O*3⋯O2^i^	0.845 (19)	1.923 (19)	2.7501 (12)	165.9 (19)
N1—H1*N*1⋯O3	0.880 (18)	2.333 (18)	2.6893 (13)	104.3 (13)
C8—H8*A*⋯O2^i^	0.991 (19)	2.565 (18)	3.2918 (16)	130.1 (13)
C14—H14*A*⋯O4	0.969 (19)	2.254 (17)	2.7752 (16)	112.6 (12)
C19—H19*A*⋯O1	0.957 (16)	2.352 (15)	2.8197 (17)	109.6 (11)

Symmetry code: (i) 
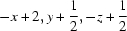
.

**Fig. 1.**
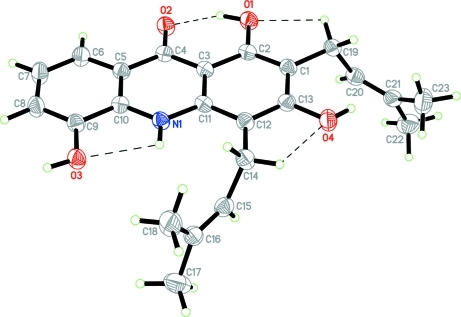
The structure of (I), showing 50% probability displacement ellipsoids and the atom-numbering scheme. Intramolecular hydrogen bonds are shown as dashed lines.

**Fig. 2.**
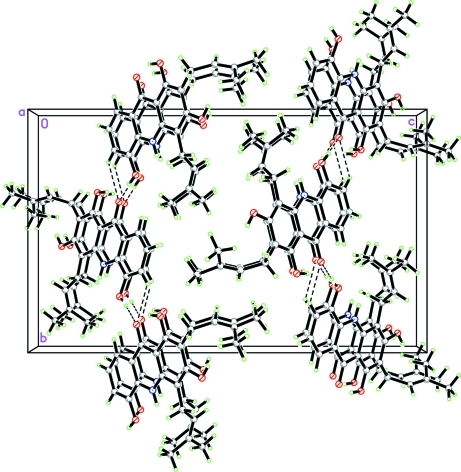
The crystal packing of (I) viewed along the a axis, showing screw chains along the [0 1 0] direction. Hydrogen bonds are shown as dashed lines.

## supplementary crystallographic information

### Comment

The title acridone alkaloid (I) known as atalaphylline (Basu & Basa, 1972), was isolated from the roots of *Atalantia monophylla* Corrêa, a mangrove plant which was collected from Trang province in the southern part of Thailand. Although (I) has been previously reported (Chantrapromma *et al*., 2010), the absolute configuration could not be determined due to insufficient anomalous dispersion from the light atoms using the data set collected with Mo radiation. The data of the same sample was recollected using Cu radiation with our newly-installed Bruker Apex-Duo CCD diffractometer and the absolute configulation was determined by making use of the large anomalous scattering of Cu Kα*X*-radiation with the Flack parameter being refined to 0.05 (13). We report herein the crystal structure of (I) with data collected using Cu radiation.

Fig. 1 shows the molecular structure of (I), bond lengths and angles are closely similar to those previously described (Chantrapromma *et al.*, 2010). (I) is chiral even though it has no chiral center because its mirror image cannot be superposed onto itself. This is due to the arrangements of the two 3-methylbut-2-enyl side-chains at atoms C1 and C12. (I) crystallized as a single enantiomer in chiral orthorhombic *P*2_1_2_1_2_1_ space group. The current structure determination represents a significant improvement compared with the structure determined from the data taken with Mo radiation and it confirmed the absolute conformation of the side-chains for (I). To be precise the two 3-methyl-2-enyl groups at C1 and C12 are attached in such a way that these two side-chains are below the acridone molecular plane indicating the (-)-anticlinal conformation with the torsion angles C2–C1–C19–C20 and C13–C12–C14–C15 are -102.65 (13) and -119.77 (33)°, respectively.

Fig. 2 shows the crystal packing of (I). Intermolecular O—H···O hydrogen bonds and weak C—H···O interactions (Table 1) linked the molecules into infinite one dimensional screw-chains along the [0 1 0] direction. These features are similar to those of the previous report by Chantrapromma *et al.* (2010) except there is an additional weak intermolecular C—H···O interaction and a π–π interaction with a Cg_1_···Cg_2_ distance of 3.7643 (7) Å (symmetry code: -1+x, y, z); Cg_1_ and Cg_2_ are the centroids of C3–C5/C10–C11/N1 and C5–C10 rings, respectively . These differences are due to the fact that all the hydrogen atoms are refined freely whereas in previous report by Chantrapromma *et al.* (2010), the hydrogen atoms were positioned geometrically and allowed to ride on their parent atoms.

### Experimental

The compound was isolated and crystal grown as reported by Chantrapromma *et al.* (2010).

### Refinement

All H atoms were located from the difference map and isotropically refined. The highest residual electron density peak is located at 0.66 Å from C3 and the deepest hole is located at 0.84 Å from H1N1. 1280 Friedel pairs were used to find the absolute configuration.

### Crystal data

**Table:**

C_23_H_25_NO_4_	*F*(000) = 808
*M**_r_* = 379.44	*D*_x_ = 1.339 Mg m^−^^3^
Orthorhombic, *P*2_1_2_1_2_1_	Cu *K*α radiation, λ = 1.54178 Å
Hall symbol: P 2ac 2ab	Cell parameters from 3145 reflections
*a* = 5.0838 (1) Å	θ = 6.1–64.9°
*b* = 15.0262 (3) Å	µ = 0.74 mm^−^^1^
*c* = 24.6412 (4) Å	*T* = 150 K
*V* = 1882.35 (6) Å^3^	Plate, brown
*Z* = 4	0.40 × 0.21 × 0.04 mm

### Data collection

**Table:**

Bruker APEX Duo CCD area-detector diffractometer	3145 independent reflections
Radiation source: sealed tube	3099 reflections with *I* > 2σ(*I*)
graphite	*R*_int_ = 0.017
φ and ω scans	θ_max_ = 64.9°, θ_min_ = 6.1°
Absorption correction: multi-scan (*SADABS*; Bruker, 2009)	*h* = −5→5
*T*_min_ = 0.755, *T*_max_ = 0.970	*k* = −17→17
11768 measured reflections	*l* = −28→28

### Refinement

**Table:**

Refinement on *F*^2^	Hydrogen site location: inferred from neighbouring sites
Least-squares matrix: full	All H-atom parameters refined
*R*[*F*^2^ > 2σ(*F*^2^)] = 0.025	*w* = 1/[σ^2^(*F*_o_^2^) + (0.0452*P*)^2^ + 0.1806*P*] where *P* = (*F*_o_^2^ + 2*F*_c_^2^)/3
*wR*(*F*^2^) = 0.068	(Δ/σ)_max_ < 0.001
*S* = 1.06	Δρ_max_ = 0.12 e Å^−^^3^
3145 reflections	Δρ_min_ = −0.10 e Å^−^^3^
354 parameters	Extinction correction: *SHELXTL* (Sheldrick, 2008), Fc^*^=kFc[1+0.001xFc^2^λ^3^/sin(2θ)]^-1/4^
0 restraints	Extinction coefficient: 0.0020 (7)
Primary atom site location: structure-invariant direct methods	Absolute structure: Flack (1983), 1280 Friedel pairs
Secondary atom site location: difference Fourier map	Flack parameter: 0.05 (13)

### Special details

**Table:**

Experimental. The crystal was placed in the cold stream of an Oxford Cryosystems Cobra open-flow nitrogen cryostat (Cosier & Glazer, 1986) operating at 150.0 (1) K.
Geometry. All esds (except the esd in the dihedral angle between two l.s. planes) are estimated using the full covariance matrix. The cell esds are taken into account individually in the estimation of esds in distances, angles and torsion angles; correlations between esds in cell parameters are only used when they are defined by crystal symmetry. An approximate (isotropic) treatment of cell esds is used for estimating esds involving l.s. planes.
Refinement. Refinement of F^2^ against ALL reflections. The weighted R-factor wR and goodness of fit S are based on F^2^, conventional R-factors R are based on F, with F set to zero for negative F^2^. The threshold expression of F^2^ > 2sigma(F^2^) is used only for calculating R-factors(gt) etc. and is not relevant to the choice of reflections for refinement. R-factors based on F^2^ are statistically about twice as large as those based on F, and R- factors based on ALL data will be even larger.

### Fractional atomic coordinates and isotropic or equivalent isotropic displacement parameters (Å^2^)

**Table:**

	*x*	*y*	*z*	*U*_iso_*/*U*_eq_	
O1	0.33887 (19)	0.33672 (5)	0.16257 (4)	0.0319 (2)	
H1O1	0.469 (4)	0.3351 (12)	0.1882 (7)	0.049 (5)*	
O2	0.72372 (18)	0.37879 (5)	0.22397 (3)	0.0312 (2)	
O3	0.90459 (19)	0.77102 (5)	0.22853 (3)	0.0318 (2)	
H1O3	0.997 (4)	0.8077 (12)	0.2462 (7)	0.044 (4)*	
O4	−0.06425 (19)	0.56721 (6)	0.06026 (4)	0.0361 (2)	
H1O4	−0.139 (5)	0.5215 (14)	0.0460 (8)	0.063 (6)*	
N1	0.6200 (2)	0.63969 (6)	0.18325 (4)	0.0264 (2)	
H1N1	0.606 (4)	0.6957 (12)	0.1732 (6)	0.038 (4)*	
C1	0.1336 (2)	0.44827 (8)	0.11088 (5)	0.0273 (3)	
C2	0.3211 (2)	0.42334 (8)	0.14856 (5)	0.0261 (3)	
C3	0.4921 (2)	0.48690 (7)	0.17304 (4)	0.0251 (3)	
C4	0.6888 (2)	0.46022 (8)	0.21176 (5)	0.0262 (3)	
C5	0.8448 (3)	0.52966 (8)	0.23719 (5)	0.0269 (3)	
C6	1.0361 (3)	0.50956 (8)	0.27663 (5)	0.0331 (3)	
H6A	1.074 (3)	0.4477 (10)	0.2852 (6)	0.030 (3)*	
C7	1.1787 (3)	0.57660 (9)	0.29998 (6)	0.0381 (3)	
H7A	1.318 (4)	0.5600 (11)	0.3267 (7)	0.045 (4)*	
C8	1.1372 (3)	0.66535 (9)	0.28512 (5)	0.0343 (3)	
H8A	1.240 (4)	0.7140 (12)	0.3021 (7)	0.057 (5)*	
C9	0.9535 (3)	0.68681 (8)	0.24628 (5)	0.0281 (3)	
C10	0.8034 (2)	0.61840 (8)	0.22173 (5)	0.0259 (3)	
C11	0.4625 (2)	0.57783 (7)	0.15855 (5)	0.0255 (2)	
C12	0.2744 (2)	0.60509 (8)	0.12029 (5)	0.0274 (3)	
C13	0.1178 (2)	0.53936 (8)	0.09727 (5)	0.0277 (3)	
C14	0.2289 (3)	0.70240 (8)	0.10748 (6)	0.0319 (3)	
H14A	0.081 (4)	0.7041 (11)	0.0827 (7)	0.049 (5)*	
H14B	0.184 (3)	0.7325 (11)	0.1402 (7)	0.040 (4)*	
C15	0.4574 (3)	0.74968 (8)	0.08153 (5)	0.0313 (3)	
H15A	0.544 (3)	0.7170 (10)	0.0517 (6)	0.041 (4)*	
C16	0.5452 (3)	0.83055 (8)	0.09368 (5)	0.0346 (3)	
C17	0.7648 (4)	0.87331 (12)	0.06276 (8)	0.0536 (4)	
H17A	0.917 (5)	0.8886 (16)	0.0890 (10)	0.087 (7)*	
H17B	0.829 (4)	0.8328 (14)	0.0332 (8)	0.065 (6)*	
H17C	0.715 (4)	0.9270 (13)	0.0451 (7)	0.053 (5)*	
C18	0.4364 (4)	0.88686 (9)	0.13869 (7)	0.0469 (4)	
H18A	0.281 (5)	0.8592 (14)	0.1596 (9)	0.071 (6)*	
H18B	0.563 (6)	0.8967 (17)	0.1648 (10)	0.096 (8)*	
H18C	0.371 (4)	0.9448 (14)	0.1242 (8)	0.063 (5)*	
C19	−0.0548 (3)	0.38053 (8)	0.08652 (5)	0.0301 (3)	
H19A	−0.048 (3)	0.3304 (11)	0.1104 (6)	0.039 (4)*	
H19B	−0.240 (4)	0.4055 (10)	0.0883 (6)	0.042 (4)*	
C20	0.0167 (3)	0.35223 (7)	0.02981 (5)	0.0312 (3)	
H20A	0.179 (3)	0.3171 (10)	0.0264 (6)	0.037 (4)*	
C21	−0.1104 (3)	0.37003 (8)	−0.01615 (5)	0.0351 (3)	
C22	−0.0091 (4)	0.33621 (11)	−0.06969 (6)	0.0509 (4)	
H22A	−0.149 (5)	0.3035 (14)	−0.0897 (8)	0.069 (6)*	
H22B	0.043 (4)	0.3915 (13)	−0.0939 (8)	0.061 (5)*	
H22C	0.179 (5)	0.2984 (15)	−0.0653 (9)	0.080 (7)*	
C23	−0.3525 (3)	0.42646 (13)	−0.02079 (7)	0.0520 (4)	
H23A	−0.490 (5)	0.3962 (16)	−0.0444 (9)	0.083 (7)*	
H23B	−0.312 (6)	0.4882 (19)	−0.0386 (10)	0.099 (8)*	
H23C	−0.438 (4)	0.4365 (11)	0.0152 (8)	0.050 (5)*	

### Atomic displacement parameters (Å^2^)

**Table:**

	*U*^11^	*U*^22^	*U*^33^	*U*^12^	*U*^13^	*U*^23^
O1	0.0368 (5)	0.0218 (4)	0.0370 (5)	−0.0033 (3)	−0.0036 (4)	−0.0006 (3)
O2	0.0342 (5)	0.0199 (4)	0.0395 (5)	0.0001 (4)	−0.0059 (4)	0.0012 (3)
O3	0.0387 (5)	0.0210 (4)	0.0357 (4)	−0.0020 (4)	−0.0080 (4)	−0.0015 (3)
O4	0.0340 (5)	0.0337 (5)	0.0406 (5)	−0.0034 (4)	−0.0116 (4)	0.0020 (4)
N1	0.0280 (5)	0.0194 (5)	0.0318 (5)	−0.0012 (4)	−0.0023 (4)	−0.0003 (4)
C1	0.0269 (6)	0.0283 (6)	0.0266 (5)	−0.0036 (5)	0.0034 (5)	−0.0033 (4)
C2	0.0280 (6)	0.0228 (5)	0.0275 (5)	−0.0009 (5)	0.0051 (5)	−0.0020 (4)
C3	0.0253 (6)	0.0234 (5)	0.0266 (5)	−0.0007 (5)	0.0032 (5)	−0.0022 (4)
C4	0.0271 (6)	0.0234 (5)	0.0280 (5)	0.0009 (4)	0.0023 (5)	−0.0008 (4)
C5	0.0286 (6)	0.0229 (6)	0.0290 (6)	−0.0007 (5)	0.0010 (5)	−0.0014 (4)
C6	0.0396 (7)	0.0238 (6)	0.0358 (6)	0.0027 (5)	−0.0084 (6)	0.0009 (5)
C7	0.0438 (8)	0.0299 (6)	0.0405 (7)	−0.0008 (6)	−0.0164 (6)	−0.0005 (5)
C8	0.0402 (7)	0.0271 (6)	0.0356 (7)	−0.0038 (5)	−0.0096 (6)	−0.0039 (5)
C9	0.0324 (7)	0.0222 (5)	0.0298 (6)	−0.0015 (5)	0.0004 (5)	−0.0027 (4)
C10	0.0261 (6)	0.0254 (6)	0.0263 (6)	0.0013 (5)	0.0012 (5)	−0.0011 (4)
C11	0.0245 (6)	0.0234 (5)	0.0285 (6)	−0.0012 (5)	0.0028 (5)	−0.0016 (4)
C12	0.0254 (6)	0.0261 (6)	0.0306 (6)	0.0000 (5)	0.0001 (5)	−0.0003 (5)
C13	0.0255 (6)	0.0295 (6)	0.0281 (5)	−0.0004 (5)	0.0012 (5)	0.0012 (5)
C14	0.0286 (7)	0.0269 (6)	0.0403 (7)	0.0007 (5)	−0.0047 (6)	0.0011 (5)
C15	0.0329 (7)	0.0300 (6)	0.0310 (6)	0.0029 (6)	−0.0039 (5)	0.0032 (5)
C16	0.0325 (7)	0.0297 (6)	0.0414 (7)	−0.0017 (5)	−0.0116 (6)	0.0106 (5)
C17	0.0408 (8)	0.0440 (8)	0.0759 (12)	−0.0058 (7)	−0.0023 (8)	0.0237 (8)
C18	0.0621 (10)	0.0288 (6)	0.0497 (8)	−0.0040 (7)	−0.0127 (8)	−0.0037 (6)
C19	0.0311 (7)	0.0291 (6)	0.0300 (6)	−0.0058 (6)	−0.0005 (5)	−0.0009 (5)
C20	0.0338 (7)	0.0245 (5)	0.0353 (6)	−0.0031 (5)	0.0022 (5)	−0.0027 (5)
C21	0.0413 (7)	0.0318 (6)	0.0323 (6)	−0.0113 (6)	−0.0007 (6)	−0.0002 (5)
C22	0.0705 (11)	0.0481 (8)	0.0341 (7)	−0.0124 (8)	0.0028 (7)	−0.0042 (6)
C23	0.0417 (8)	0.0680 (11)	0.0465 (9)	0.0000 (8)	−0.0128 (7)	−0.0005 (8)

### Geometric parameters (Å, °)

**Table:**

O1—C2	1.3497 (15)	C12—C14	1.5137 (16)
O1—H1O1	0.92 (2)	C14—C15	1.5045 (18)
O2—C4	1.2725 (14)	C14—H14A	0.97 (2)
O3—C9	1.3617 (14)	C14—H14B	0.954 (16)
O3—H1O3	0.845 (19)	C15—C16	1.3285 (18)
O4—C13	1.3651 (15)	C15—H15A	0.986 (16)
O4—H1O4	0.86 (2)	C16—C17	1.497 (2)
N1—C10	1.3679 (16)	C16—C18	1.501 (2)
N1—C11	1.3695 (15)	C17—H17A	1.03 (3)
N1—H1N1	0.880 (17)	C17—H17B	1.00 (2)
C1—C2	1.3825 (18)	C17—H17C	0.95 (2)
C1—C13	1.4114 (17)	C18—H18A	1.03 (2)
C1—C19	1.5209 (16)	C18—H18B	0.92 (3)
C2—C3	1.4254 (16)	C18—H18C	1.00 (2)
C3—C11	1.4203 (16)	C19—C20	1.5051 (17)
C3—C4	1.4393 (17)	C19—H19A	0.957 (16)
C4—C5	1.4525 (16)	C19—H19B	1.015 (19)
C5—C10	1.4026 (17)	C20—C21	1.3311 (19)
C5—C6	1.4078 (18)	C20—H20A	0.982 (17)
C6—C7	1.3679 (19)	C21—C23	1.499 (2)
C6—H6A	0.973 (15)	C21—C22	1.505 (2)
C7—C8	1.3990 (19)	C22—H22A	0.99 (2)
C7—H7A	0.997 (18)	C22—H22B	1.06 (2)
C8—C9	1.3754 (18)	C22—H22C	1.12 (3)
C8—H8A	0.99 (2)	C23—H23A	1.02 (3)
C9—C10	1.4159 (17)	C23—H23B	1.05 (3)
C11—C12	1.4041 (17)	C23—H23C	0.999 (19)
C12—C13	1.3895 (17)		
			
C2—O1—H1O1	104.5 (11)	C12—C14—H14A	106.0 (10)
C9—O3—H1O3	109.9 (12)	C15—C14—H14B	108.8 (10)
C13—O4—H1O4	109.2 (14)	C12—C14—H14B	108.6 (10)
C10—N1—C11	123.22 (10)	H14A—C14—H14B	109.5 (14)
C10—N1—H1N1	118.3 (11)	C16—C15—C14	126.55 (13)
C11—N1—H1N1	118.5 (11)	C16—C15—H15A	118.3 (10)
C2—C1—C13	117.48 (11)	C14—C15—H15A	115.1 (9)
C2—C1—C19	121.19 (11)	C15—C16—C17	121.91 (15)
C13—C1—C19	121.29 (11)	C15—C16—C18	123.95 (13)
O1—C2—C1	118.64 (10)	C17—C16—C18	114.14 (14)
O1—C2—C3	119.81 (11)	C16—C17—H17A	109.5 (14)
C1—C2—C3	121.55 (11)	C16—C17—H17B	110.5 (12)
C11—C3—C2	118.26 (10)	H17A—C17—H17B	110.4 (19)
C11—C3—C4	120.55 (10)	C16—C17—H17C	113.6 (12)
C2—C3—C4	121.18 (10)	H17A—C17—H17C	107.3 (17)
O2—C4—C3	121.43 (11)	H17B—C17—H17C	105.5 (16)
O2—C4—C5	120.84 (11)	C16—C18—H18A	115.1 (12)
C3—C4—C5	117.72 (10)	C16—C18—H18B	110.4 (17)
C10—C5—C6	119.67 (11)	H18A—C18—H18B	104.6 (18)
C10—C5—C4	118.94 (11)	C16—C18—H18C	110.5 (11)
C6—C5—C4	121.39 (11)	H18A—C18—H18C	105.9 (17)
C7—C6—C5	119.90 (12)	H18B—C18—H18C	110 (2)
C7—C6—H6A	120.5 (9)	C20—C19—C1	113.79 (10)
C5—C6—H6A	119.5 (9)	C20—C19—H19A	109.9 (9)
C6—C7—C8	120.79 (12)	C1—C19—H19A	105.1 (10)
C6—C7—H7A	118.0 (10)	C20—C19—H19B	111.7 (9)
C8—C7—H7A	121.2 (10)	C1—C19—H19B	108.6 (9)
C9—C8—C7	120.54 (12)	H19A—C19—H19B	107.4 (14)
C9—C8—H8A	118.7 (11)	C21—C20—C19	128.01 (13)
C7—C8—H8A	120.8 (11)	C21—C20—H20A	116.2 (9)
O3—C9—C8	124.42 (11)	C19—C20—H20A	115.8 (9)
O3—C9—C10	116.04 (11)	C20—C21—C23	125.26 (13)
C8—C9—C10	119.53 (11)	C20—C21—C22	120.79 (14)
N1—C10—C5	120.86 (11)	C23—C21—C22	113.91 (14)
N1—C10—C9	119.58 (11)	C21—C22—H22A	110.9 (12)
C5—C10—C9	119.56 (11)	C21—C22—H22B	108.4 (10)
N1—C11—C12	119.91 (10)	H22A—C22—H22B	106.7 (15)
N1—C11—C3	118.64 (11)	C21—C22—H22C	112.3 (11)
C12—C11—C3	121.44 (10)	H22A—C22—H22C	114.1 (16)
C13—C12—C11	117.19 (11)	H22B—C22—H22C	103.9 (16)
C13—C12—C14	120.93 (11)	C21—C23—H23A	110.8 (14)
C11—C12—C14	121.73 (11)	C21—C23—H23B	111.8 (17)
O4—C13—C12	116.30 (11)	H23A—C23—H23B	107 (2)
O4—C13—C1	119.64 (11)	C21—C23—H23C	112.1 (11)
C12—C13—C1	124.04 (11)	H23A—C23—H23C	106.0 (17)
C15—C14—C12	115.26 (11)	H23B—C23—H23C	108.9 (17)
C15—C14—H14A	108.6 (10)		
			
C13—C1—C2—O1	179.51 (10)	O3—C9—C10—C5	179.04 (11)
C19—C1—C2—O1	1.60 (17)	C8—C9—C10—C5	−0.24 (18)
C13—C1—C2—C3	0.19 (17)	C10—N1—C11—C12	178.53 (10)
C19—C1—C2—C3	−177.72 (10)	C10—N1—C11—C3	−0.68 (17)
O1—C2—C3—C11	−178.11 (10)	C2—C3—C11—N1	177.81 (10)
C1—C2—C3—C11	1.20 (17)	C4—C3—C11—N1	−1.72 (16)
O1—C2—C3—C4	1.42 (16)	C2—C3—C11—C12	−1.39 (16)
C1—C2—C3—C4	−179.27 (11)	C4—C3—C11—C12	179.08 (11)
C11—C3—C4—O2	−177.79 (11)	N1—C11—C12—C13	−179.03 (11)
C2—C3—C4—O2	2.69 (17)	C3—C11—C12—C13	0.16 (17)
C11—C3—C4—C5	3.05 (16)	N1—C11—C12—C14	−3.44 (17)
C2—C3—C4—C5	−176.47 (10)	C3—C11—C12—C14	175.74 (11)
O2—C4—C5—C10	178.75 (11)	C11—C12—C13—O4	179.87 (10)
C3—C4—C5—C10	−2.09 (16)	C14—C12—C13—O4	4.25 (17)
O2—C4—C5—C6	−1.00 (18)	C11—C12—C13—C1	1.35 (18)
C3—C4—C5—C6	178.17 (12)	C14—C12—C13—C1	−174.27 (11)
C10—C5—C6—C7	0.6 (2)	C2—C1—C13—O4	180.00 (10)
C4—C5—C6—C7	−179.67 (12)	C19—C1—C13—O4	−2.10 (17)
C5—C6—C7—C8	−0.1 (2)	C2—C1—C13—C12	−1.53 (18)
C6—C7—C8—C9	−0.6 (2)	C19—C1—C13—C12	176.37 (11)
C7—C8—C9—O3	−178.45 (13)	C13—C12—C14—C15	−119.77 (13)
C7—C8—C9—C10	0.8 (2)	C11—C12—C14—C15	64.81 (16)
C11—N1—C10—C5	1.65 (17)	C12—C14—C15—C16	−136.97 (13)
C11—N1—C10—C9	−178.35 (11)	C14—C15—C16—C17	−176.18 (13)
C6—C5—C10—N1	179.57 (11)	C14—C15—C16—C18	3.8 (2)
C4—C5—C10—N1	−0.18 (17)	C2—C1—C19—C20	−102.65 (13)
C6—C5—C10—C9	−0.43 (18)	C13—C1—C19—C20	79.52 (15)
C4—C5—C10—C9	179.82 (11)	C1—C19—C20—C21	−111.01 (15)
O3—C9—C10—N1	−0.96 (16)	C19—C20—C21—C23	2.4 (2)
C8—C9—C10—N1	179.75 (11)	C19—C20—C21—C22	179.80 (13)

### Hydrogen-bond geometry (Å, °)

**Table:**

*D*—H···*A*	*D*—H	H···*A*	*D*···*A*	*D*—H···*A*
O1—H1O1···O2	0.915 (19)	1.699 (19)	2.5528 (13)	154.1 (17)
O3—H1O3···O2^i^	0.845 (19)	1.923 (19)	2.7501 (12)	165.9 (19)
N1—H1N1···O3	0.880 (18)	2.333 (18)	2.6893 (13)	104.3 (13)
C8—H8A···O2^i^	0.991 (19)	2.565 (18)	3.2918 (16)	130.1 (13)
C14—H14A···O4	0.969 (19)	2.254 (17)	2.7752 (16)	112.6 (12)
C19—H19A···O1	0.957 (16)	2.352 (15)	2.8197 (17)	109.6 (11)

Symmetry codes:  (i) −*x*+2, *y*+1/2, −*z*+1/2.
